# Variation in Bat Guano Bacterial Community Composition With Depth

**DOI:** 10.3389/fmicb.2018.00914

**Published:** 2018-05-11

**Authors:** Molli M. Newman, Laura N. Kloepper, Makenzie Duncan, John A. McInroy, Joseph W. Kloepper

**Affiliations:** ^1^Department of Biology, LaGrange College, LaGrange, GA, United States; ^2^Department of Biology, Saint Mary’s College, Notre Dame, IN, United States; ^3^Department of Entomology and Plant Pathology, Auburn University, Auburn, AL, United States

**Keywords:** *Tadarida brasiliensis*, bats, guano, bacterial communities, bacterial diversity, *Chiroptera*

## Abstract

Bats are known to be reservoirs for a variety of mammalian pathogens, including viruses, fungi, and bacteria. Many of the studies examining the microbial community inhabiting bats have investigated bacterial taxa found within specific bat tissues and isolated bat guano pellets, but relatively few studies have explored bacterial diversity within bat guano piles. In large bat caves, bat guano can accumulate over time, creating piles several meters deep and forming complex interactions with coprophagous organisms in a habitat with low light and oxygen. As the guano decays, the nutrient composition changes, but the bacterial communities deep within the pile have not been characterized. Here, we assess the bacterial communities across varying depths within the guano pile using both culture-independent and culture-dependent methods. We found that although similar taxa are found throughout the guano pile, the relative abundances of taxa within the pile shift, allowing certain taxa to dominate the bacterial community at varying depths. We also identified potential bacterial functions being performed within the bat guano as various depths within the pile and found little variation in terms of the dominant predicted functions, suggesting that although the relative abundances of bacterial taxa are changing, the functions being performed are similar. Additionally, we cultured 15 different bacterial species, including 2 not present in our culture-independent analysis, and discuss the pathogenicity potential of these taxa. This study represents the first characterization of the bacterial community from the extreme environment within a bat guano pile and demonstrates the potential for bat caves as resources for identifying new bacterial species.

## Introduction

Bats are considered reservoirs for several microbial pathogens, including viruses (Leroy et al., 2005; Calisher et al., 2006; Wang and Eaton, 2007; Towner et al., 2009), fungi (DiSalvo et al., 1969; Mok et al., 1982), and bacteria (Veikkolainen et al., 2014; Banskar et al., 2016). Recent advances in metagenomic sequencing have allowed for a preliminary understanding of the bat microbial community from saliva and urine (Dietrich et al., 2017), the intestine (Banskar et al., 2014; Carrillo-Araujo et al., 2015), and the colon (Phillips et al., 2012).

Although most information on pathogens from bat guano comes from isolated guano pellets, the guano piles from within large cave colonies may contain even greater numbers of pathogens or possible new bacterial species (Chroňáková et al., 2009; De Mandal et al., 2015). Bat guano piles, especially maternal roosts located in the American Southwest, represent unique ecological habitats. These habitats are exposed to low levels of UV radiation, and in the summers these caves fill with millions of bats, reach internal temperatures up to 43°C (Herreid, 1963), and have high gaseous ammonia levels (McFarlane et al., 1995). As a result of the bat residence, guano constantly falls from the cave ceiling and accumulates on the cave floor, piling and forming a large reservoir of organic material in a unique micro-habitat for microbial growth (Fenolio et al., 2006). The chemical and physical properties of bat guano vary over space and time. Guano on the surface of the pile is fresher, moister, more alkaline, and contains more nitrogen than the guano deeper in the pile (Bernath and Kunz, 1981; Ferreira and Martins, 1999; Chroňáková et al., 2009). These depth-related changes in properties could, therefore, lead to differences in bacterial composition throughout the guano pile.

Bacterial communities have begun to be characterized from bat guano piles within caves, but previous studies have only investigated the surface of the guano pile or sampled from caves with small bat populations and low ambient temperatures (Chroňáková et al., 2009; De Mandal et al., 2015). Because *in vitro* studies of decaying bat guano indicate higher diversity of bacterial communities in decaying guano relative to fresher guano (Banskar et al., 2016), and the caves themselves harbor unique geomicrobial interactions (Boston et al., 2001; Northup and Lavoie, 2001), characterizing the bacterial communities from decaying guano *in situ* (i.e., in the guano pile) is important for understanding the microbial community, including potential pathogens, in this unique habitat.

In this study, we sampled three regions of bat guano from a large maternal colony of the insectivorous *Tadarida brasiliensis* in southern New Mexico: fresh guano, collected as bats were returning to their roost after nightly foraging, surface pile guano, collected from the top of the guano pile, and decaying guano, collected from within the guano pile. From these samples, we characterized the bacterial community via both culture-independent analysis, where we quantified differences in relative abundance on the family and genus across treatments, and culture-dependent analysis, where we cultured bacteria and sequenced them for identification. This study is the first characterization and comparison of the bacterial community in bat guano across varying levels of decay.

## Materials and Methods

### Culture-Independent Analysis

Guano was collected for 7 days between June 21 and June 27, 2016 from a lava tube cave structure located in Sierra County, NM, United States. The cave housed an active maternal colony of approximately 400,000 *Tadarida brasiliensis* (Kloepper et al., 2016). Each day we collected three treatments of guano corresponding to different levels of decay: fresh, surface guano pile, and subsurface guano pile.

Fresh guano was collected by laying a sterile plastic sheet on the ground outside the opening of the cave each morning to capture excreted guano as bats returned after nightly foraging. The fresh guano was then hand picked off the plastic using sterilized gloves and placed into sterile Whirl-Pak bags. Surface and subsurface (0.5 m deep) guano pile samples were collected from inside the cave at sunrise using sterile gloves and Whirl-Pak bags. Samples were collected directly below the highest density grouping of bats. All personnel entering the cave had the rabies pre-exposure vaccine, wore sterile Tyvek suits and full-face respirators, and followed all National White-Nose Syndrome decontamination protocols.

Samples were kept at 4°C during the sampling period and shipped on ice via overnight shipping to Auburn University. Upon arrival, samples were kept at -80°C until DNA extraction to prevent any degradation of genomic DNA prior to extractions. Genomic DNA within bat guano samples was extracted from 50 mg of guano using the E.Z.N.A.^®^ Omega Bio-tek Stool DNA extraction kit. DNA concentration of each sample was measured using the Qubit dsDNA BR Assay kit (Thermo Fisher Scientific, United States).

The V4 hypervariable region of the 16S rRNA gene was amplified using the HotStarTaq Plus Master Mix Kit (Qiagen, United States) using the 515F/806R primer pair (Caporaso et al., 2012) with a barcode on the forward primer. The following conditions were used for this amplification reaction: 94°C for 3 min, followed by 28 cycles of 94°C for 30 s, 53°C for 40 s, and 72°C for 1 min, after which a final elongation step at 72°C for 5 min was performed.

Following amplification, PCR products were verified on a 2% agarose gel. Samples were pooled together in equimolar concentrations and purified using calibrated Ampure XP beds. The pooled and purified PCR products were then used to construct an Illumina DNA library. Sequencing was performed at MR DNA^1^ (Shallowater, TX, United States) on a MiSeq following the manufacturer’s guidelines.

Sequence data were processed using MR DNA analysis pipeline (MR DNA, Shallowater, TX, United States). In summary, sequences were joined, barcodes were removed, and sequences were filtered to remove those < 150 bp in length and having ambiguous base calls. Operational taxonomic units (OTUs) were generated using uclust, and the relative OTU abundance of each OTU was estimated. Chimeras were removed by usearch v6.1 using the Quantitative Insights Into Microbial Ecology (QIIME) pipeline (Caporaso et al., 2010b). OTUs were defined by clustering at 3% divergence (97% similarity). Final OTUs were taxonomically classified by uclust using BLASTn against a curated database derived from RDPII^2^ and NCBI^3^. The resulting OTU table was rarified to a depth of 79,900 sequences based on the sample with the fewest number of sequences generated. For phylogenetic calculations, sequences were aligned using PyNAST (Caporaso et al., 2010a), and a phylogenetic tree was constructed using FastTree (Price et al., 2009).

To compare beta diversity among sample sites, unweighted and weighted UniFrac metrics were calculated. These values were used as input into a principal coordinates analysis to visualize similarity/dissimilarity among the bacterial communities within the various sampling sites. The weighted UniFrac values were also used as input to conduct an Adonis test (Anderson, 2001) in QIIME.

Alpha diversity metrics, including the number of observed OTUs, Chao1 richness estimate, Shannon’s Index, Simpson’s evenness, and Faith’s Phylogenetic Diversity, were calculated using the QIIME pipeline (Caporaso et al., 2010b). These values were compared between sample sites using a non-parametric two-sample *t*-test with Monte Carlo permutations. OTU frequencies were compared using an ANOVA to determine whether significant differences in OTU abundance existed among sampling sites.

Phylogenetic investigation of communities by reconstruction of unobserved states (PICRUSt) analysis (v1.1.3; Langille et al., 2013) was performed to further explore potential bacterial functions within the bat guano samples. In PICRUSt, the OTU table produced above was normalized by copy number to correct for multiple 16S copy number and metagenome predictions were made, generating KEGG Ortholog (KO) abundances for each sample. Functional predictions were then assigned up to KO tier 3.

### Culture-Dependent Analysis

Guano was collected from the same location as the Culture-Independent Analysis on May 25–27, 2017, from four distinct sources: fresh, surface, subsurface 1 (at 77 cm), and subsurface 2 (at 122 cm). Fresh guano was collected by obtaining samples off a plastic sheet positioned near the entrance of the cave before the bats returned from foraging. Upon return, the bats defecated on this sheet, and samples were quickly harvested. The surface samples were taken from the top of the main guano pile within the cave. Subsurface samples were collected using a 1′′× 36′′ Plated Replaceable Tip Soil Probe with 24′′ Slot and a 36′′ extension (AMS, American Falls, ID, United States), yielding two distinct subsurface samples at 77 cm and 122 cm. The probe was sterilized with ethanol before each sample collection. Samples were stored in sterile Whirl-Pak bags and shipped overnight to Auburn University on ice where they were stored in a refrigerator at 4°C for 5 days before further analysis.

A 0.065-g sample of guano from each treatment (equivalent to 12 guano pellets) was randomly selected for bacterial culturing. The guano was mixed with 30.0 mL of sterile water and serially diluted to 10^-2^, 10^-4^, and 10^-6^. A total of 50 μL from each dilution was then spread-plated on 20% tryptic soy agar (TSA, Becton, Dickinson and Company, Franklin Lakes, NJ, United States), blood agar (BA, Ward’s Science, Rochester, NY, United States), and a specially composed medium we designated bat guano medium (BGM). BGM contained several carbon sources and ammonium chloride to mimic the nitrogen-rich components of the droppings as well as the atmosphere of the cave (see **Supplementary Table S1** for recipe). Plates were incubated at 28°C for 3 days to allow for sufficient growth. Distinct colonies were transferred to fresh agar plates to allow for pure colony growth. Pure cultures were maintained for 48 h at 28°C and then transferred to glycerol stocks (TSA amended with 30% glycerol) and kept in ultracold storage. The isolated strains were identified using PCR amplification of the 16S rRNA gene. PCR was conducted with EconoTaq^®^ Plus Green 2X Master Mix (Lucigen) and the following universal primers, 8F and 1492R (Lane, 1991). Thermal cycler settings were 95°C for 5 min, followed by 31 cycles of: 94°C for 1 min, 57°C for 45 s, 70°C for 2 min, and a final extension at 70°C for 8 min. PCR products were checked for amplicons of approximately 1.5 kb by gel electrophoresis in a 1% agarose gel in TBE buffer, subjected to 100 V for 27 min and a 2-Kbp DNA ladder for comparison. Agarose bands were visualized using a C300 imaging system (Azure Biosystems). All strains that generated an amplicon in the expected range (1400–1500 bp) were sent to Molecular Cloning Laboratories (MCLAB) in South San Francisco, CA, United States, for 16S rRNA gene sequencing using 8F, 1492R, and 907R primers (Lane, 1991). Raw sequence data were manually edited in Geneious to create a near-complete consensus sequence for each strain. Consensus sequences were subjected to analysis at https://www.ezbiocloud.net/ for taxonomic placement. Taxonomic results were then researched (online) to determine whether the species found in the samples were known to cause disease or appear in clinical samples.

## Results

### Sequencing Summary

Following filtering, a total of 3,194,104 bacterial 16S rRNA gene sequences were obtained with a range of 79,936–199,876 sequences per sample and a mean of 152,100 sequences per sample. All sequences obtained in this study were submitted to the NCBI Sequence Read Archive (SRA) and are available under the study accession number PRJNA429934.

### Bat Guano Bacterial Community Diversity

**Table 1** shows the diversity indices of bacterial communities for each sampling site. Chao1, Observed OTUs, and Faith’s Phylogenetic Diversity show similar diversity among fresh, surface, and subsurface bat guano bacterial communities. Significant differences in diversity among the three sampling locations were observed for Simpson’s Evenness and Shannon’s Diversity. Simpson’s evenness estimates showed significant differences among the sampling locations with fresh bat guano having the highest bacterial community evenness (0.008 ± 0.002) and subsurface bat guano having significantly less bacterial community evenness (0.005 ± 0.0002) than both fresh and surface bat guano (*p* ≤ 0.01). Shannon’s diversity was significantly higher in the fresh bat guano bacterial community (3.66 ± 0.21) compared to the subsurface bat guano bacterial community (3.03 ± 0.18) (*p* ≤ 0.05).

**Table 1:** Alpha diversity indices of bacterial communities for each sample site.

Diversity metric	Fresh	Surface	Subsurface
Chao1	928.60 ± 103.40^a^	900.13 ± 58.49^a^	913.81 ± 85.75^a^
Observed OTUs	624.80 ± 90.01^a^	603.14 ± 34.79^a^	609.84 ± 54.20^a^
Faith’s Phylogenetic Distance	44.70 ± 6.97^a^	43.45 ± 2.31^a^	43.10 ± 3.58^a^
Shannon	3.66 ± 0.21^a^	3.35 ± 0.30^ab^	3.03 ± 0.18^b^
Simpson’s Evenness	0.0082 ± 0.0027^a^	0.0064 ± 0.0013^a^	0.0052 ± 0.0025^b^


*Values indicate mean ± 1 SD, and letters represent sample groupings based on similarity among the three treatments at the *p* ≤ 0.05 level.*

**FIGURE 1:**
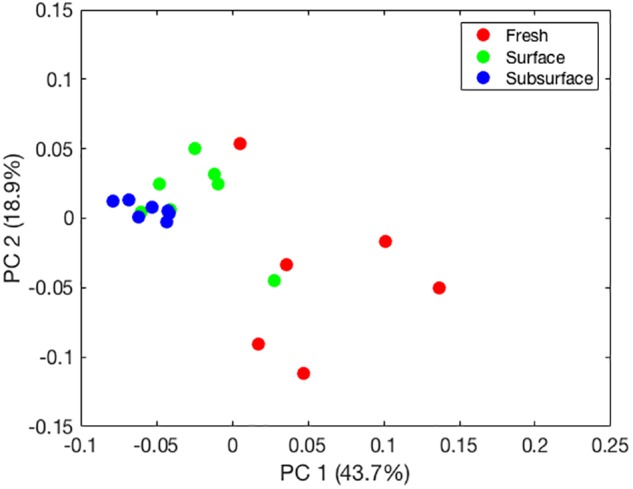
Principal component plot of bat guano bacterial community composition based on weighted UniFrac distances generated for fresh, surface, and subsurface bat guano.

Adonis tests showed significant differences among sampling locations based on weighted UniFrac distances (*p* = 0.001). A principal component analysis (PCA) of these distances (**Figure 1**) revealed separation of the bat guano bacterial communities along a gradient corresponding to sampling location (e.g., fresh to subsurface), which explained 43.7% of the variation in the bacterial communities. In addition, variability among principal components decreased from fresh bat guano to subsurface bat guano samples.

### Bat Guano Bacterial Community Composition

**FIGURE 2:**
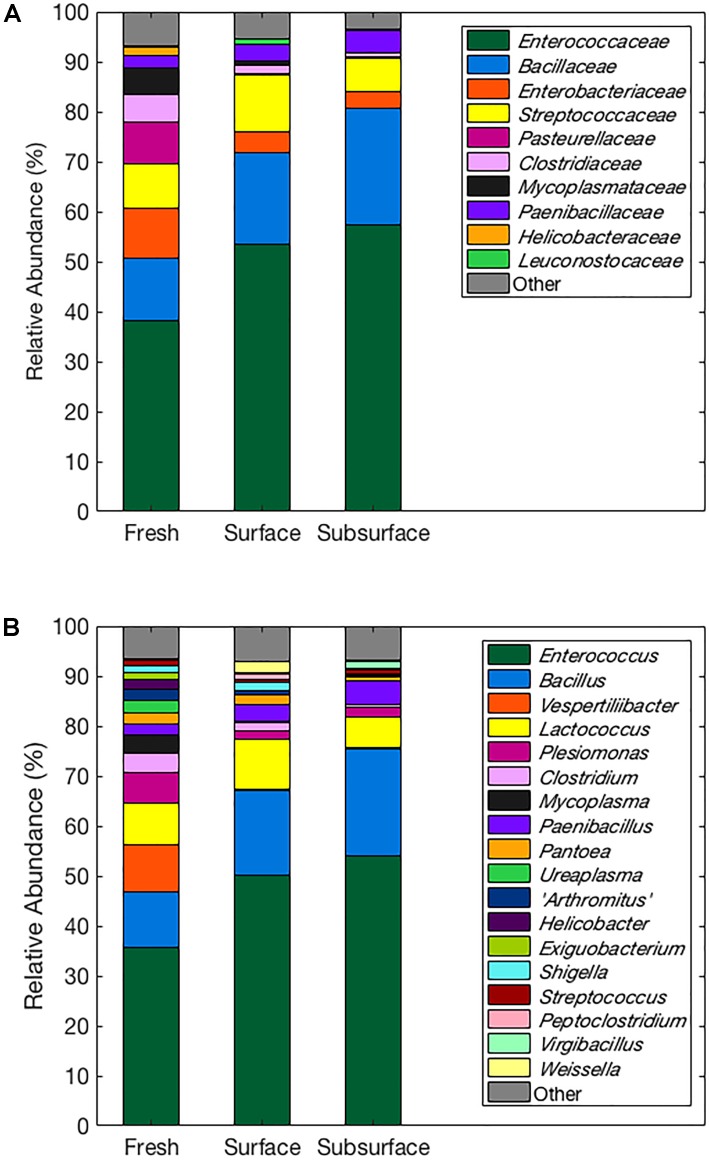
Relative abundances of bacterial taxa at the family **(A)** and genus **(B)** levels across sampling locations. All families or genera with 1% or more mean relative abundances from each sample treatment are represented by name, and families and genera with less than 1% mean relative abundances are grouped into the “other” category.

**Figure 2** represents the relative abundances of bacterial taxa at the family (**Figure 2A**) and genus (**Figure 2B**) levels across sampling locations. All bat guano sampling locations shared some similarities in terms of the bacterial families found within them. For example, all locations contained bacterial taxa from the *Enterococcaceae* and *Bacillaceae* families which were the dominant members of these communities. However, their abundances differed among the three locations with subsurface having the highest relative abundance of both *Enterococcaceae* (57.44%; dominant genus: *Enterococcus*) and *Bacillaceae* (23.17%; dominant genus: *Bacillus*) compared to 53.35 and 18.36% in surface and 38.21 and 12.60% in fresh, for *Enterococcaceae* and *Bacillaceae*, respectively. In contrast to this, members of the families *Enterobacteriaceae* (dominant genus: *Plesiomonas*) and *Pasteurellaceae* (dominant genus: *Vespertiliibacter*) had a higher relative abundance in fresh bat guano (9.77 and 8.37%, respectively) and tended to decrease in abundance in surface (4.37 and 0.12%) and subsurface (3.47 and 0.32%) bat guano.

A total of 61 OTUs were significantly different in frequency among the three sampling locations (*p* ≤ 0.05; see **Supplementary Table S2**). Many of these (47 out of 61) were identified as being in the class *Bacilli* and tended to increase in frequency from fresh bat guano to subsurface bat guano. These *Bacilli* came from the families *Bacillaceae*, *Paenibacillaceae*, *Planococcaceae*, *Enterococcaceae*, and *Streptococcaceae*. Other bacterial classes observed to be different in frequency among the three sampling locations were *Actinobacteria* and *Gamma-Proteobacteria. Actinobacteria* increased in OTU frequency from fresh to subsurface. *Gamma-Proteobacteria* from the families *Pasteurellaceae* and *Moraxellaceae* were found to change in frequency among the sampling locations with *Pasteurellaceae* decreasing in frequency from fresh to subsurface and *Moraxellaceae* increasing in frequency from fresh to subsurface.

### Functional Characterization of Bat Guano Bacterial Communities

The greatest number of genes (>45%) that were assigned a function included proteins involved in “metabolism” among tier 1 KO categories based on PICRUSt analysis (**Table 2**). In addition, “environmental information processing” and “genetic information processing” contributed to ∼19 and ∼17%, respectively, among the three sample types (**Table 2**). A small percentage (0.85–0.87%) of genes encoding proteins involved in human diseases was also identified in all three sample types. Overall, PICRUSt analysis showed little variation in the relative abundance of second- and third-tier KO functional groupings among the three bat guano depth treatments.

**Table 2:** Percentages of predicted sequences assigned to tier 2 KO categories.

Functions^∗^	Fresh	Deep	Surface
**Cell Processes**^†^	2.05	1.79	1.74
Transport and Catabolism	0.18	0.19	0.18
Cell Growth and Death	0.39	0.42	0.42
Cell Motility	1.48	1.19	1.14
**Environmental Information Processing**	19.25	19.25	19.08
Membrane Transport	17.03	17.09	16.96
Signal Transduction	2.02	1.92	1.89
Signaling Molecules and Interaction	0.22	0.26	0.25
**Genetic Information Processing**	16.98	17.20	17.34
Transcription	3.03	3.02	3.00
Translation	4.60	4.75	4.81
Folding, Sorting and Degradation	2.02	1.91	1.94
Replication and Repair	7.35	7.54	7.60
**Human Diseases**	0.87	0.85	0.87
Cancers	0.06	0.04	0.04
Immune System Diseases	0.06	0.06	0.06
Infectious Diseases	0.51	0.50	0.51
Metabolic Diseases	0.08	0.08	0.08
Neurodegenerative Diseases	0.17	0.18	0.18
**Metabolism**	45.03	45.70	45.57
Amino Acid Metabolism	8.28	8.15	8.14
Biosynthesis of Other Secondary Metabolites	0.73	0.73	0.72
Carbohydrate Metabolism	11.57	12.24	12.11
Energy Metabolism	4.64	4.53	4.55
Enzyme Families	1.97	1.90	1.90
Glycan Biosynthesis and Metabolism	1.85	1.61	1.71
Lipid Metabolism	2.98	3.11	3.06
Metabolism of Cofactors and Vitamins	3.54	3.29	3.37
Metabolism of Other Amino Acids	1.61	1.60	1.61
Metabolism of Terpenoids and Polyketides	1.65	1.79	1.75
Nucleotide Metabolism	3.75	3.97	3.98
Xenobiotics Biodegradation and Metabolism	2.52	2.83	2.71
**Organismal Systems**	0.41	0.35	0.36
Digestive System	0.03	0.02	0.02
Endocrine System	0.16	0.15	0.15
Excretory System	0.02	0.01	0.01
Immune System	0.04	0.03	0.03
Nervous System	0.05	0.05	0.05
Environmental Adaptation	0.10	0.09	0.09


*^∗^Functional categories for which no reads were assigned are omitted. ^†^Bolded functions represent tier 1 functional categories.*

### Culture Analysis

A total of 100 bacterial samples were collected and subjected to 16S rRNA gene sequencing for identification. These 100 bacterial isolates represented 15 distinct bacterial species (**Table 3**). Of the 15 species, one species (*Filibacter limicola*) yielded a low-enough similarity index using the EzBioCloud software (98.51%) to cast doubt on the accuracy of identification. We refer to this isolate as *Filibacter limicola*, but the strain is now being subjected to further analysis to see if new-species status is warranted.

**Table 3:** List of cultured bacterial species, the sampling location and media type for each species, clinical relevance, and maximum similarity of the 16S rRNA sequence using EzBioCloud software.

Bacterial species	Location found	Media	Clinically relevant?	Maximum similarity
*Microbacterium oxydans*	Fresh, Surface, Subsurface I, Subsurface II	20% TSA, BA	Y	100%
*Enterococcus faecalis*	Fresh, Surface	20% TSA, BA	Y	100%
*Lactococcus garvieae*	Surface	20% TSA	Y	100%
*Enterobacter asburiae*	Surface	20% TSA	Y	99.6%
*Microbacterium maritypicum*	Fresh, Surface, Subsurface I, Subsurface II	20% TSA, BA, BGM	N	100%
*Bacillus safensis*	Fresh, Surface, Subsurface I, Subsurface II	20% TSA, BA	N	100%
*Bacillus velezensis*	Fresh, Surface, Subsurface I, Subsurface II	20% TSA, BA, BGM	N	99.9%
*Pantoea vagans*	Fresh	BA	N	99.8%
*Staphylococcus lugdunensis*	Fresh	BA	Y	100%
*Weissella confusa*	Fresh	BGM	Y	100%
*Lactococcus lactis*	Surface	20% TSA	N	99.9%
*Filibacter limicola*	Subsurface I	BA	N	98.5%
*Deinococcus radiopugnans*	Fresh	20% TSA	N	99.9%
*Streptomyces cremeus*	Fresh	20% TSA	N	99.9%
*Streptococcus rubneri*	Subsurface II	20% TSA	N	99.6%


Some of the isolates, such as *Microbacterium maritypicum*, were able to grow on all three of the media types while others, such as *Weissella confusa*, were limited to growth on only one medium. Of our 15 cultured species, 5 were identified in fresh guano (*Pantoea vagans*, *Staphylococcus lugdunensis*, *Weissella confusa*, *Deinococcus radiopugnans*, and *Streptomyces cremeus*), 3 in surface guano (*Lactococcus garvieae*, *Enterobacter asburiae*, and *Lactococcus lactis*), 1 from 77 cm depth (*Filibacter limicola*), and 1 from 122 cm depth (*Streptococcus rubneri*), while the remaining species were found in multiple or all of the guano sources (*Microbacterium oxydans*, *Enterococcus faecalis*, *Microbacterium maritypicum*, *Bacillus safensis*, and *Bacillus velezensis*). Six species were deemed clinically significant based on their published ability to cause disease in humans (**Table 3**). These include *Enterococcus faecalis* (found in fresh and surface samples), *Lactococcus garvieae* and *Enterobacter asburiae* (found in surface samples only), *Staphylococcus lugdunensis* and *Weissella confusa* (found in fresh samples only), and *Microbacterium oxydans* (found in all sources).

## Discussion

Our results represent the first characterization of the bacterial community from the extreme environment within a bat guano pile. Specifically, we characterized the bacterial community of bat guano across varying levels of depth and decay within the cave and cultured bacterial isolates from fresh, surface pile, and deep (subsurface) guano. The use of both culture-dependent and culture-independent approaches allowed us to examine overall bacterial community composition differences among the various depths while also allowing for identification of several culturable isolates. This dual-method approach thus provides an initial dataset to examine micro-habitat differences from within the extreme habitat of a guano pile within a large bat cave.

In general, both approaches identified similar bacterial taxa at the various depths. Eight of our cultured species are found within six of the dominant genera identified from the culture-independent bacterial community composition analysis, including *Bacillus*, *Enterococcus*, *Lactococcus*, *Pantoea*, *Streptococcus*, and *Weissella* (**Figure 2B** and **Table 3**). Four cultured species also matched genera that were identified in smaller quantities from our culture-independent analysis, including *Deinococcus*, *Enterobacter*, *Streptomyces*, and *Staphylococcus*. In addition, five of the genera identified using a culture-independent approach (**Figure 2**) have been previously identified from frugivorous guano in India (*Rousettus leschenaultii*): *Weissella*, *Lactococcus*, *Enterococcus*, *Bacillus*, and *Arthrobacter* (Banskar et al., 2016). These species are present in both insectivorous and frugivorous bats across continents, which suggest they may be prevalent in the guano of bats across regions and foraging habits.

There were, however, three cultured species not present, even in trace amounts, in our community composition (culture-independent) analysis, including *Microbacterium oxydans*, *Microbacterium maritypicum*, and *Filibacter limicola.* The guano samples for the community composition were collected one year before the culture analysis samples, so the differences in species between our two analysis techniques indicates that the bacterial community within the guano pile may vary spatially or temporally. We predict that samples that differ further in time and/or location would also yield additional species.

In terms of the differences in bat guano bacterial community composition along the depth gradient studied here, our results showed clear changes in bacterial community beta diversity (Shannon) and evenness (Simpson), both of which decreased with depth (**Table 1**). However, no significant changes were observed in alpha diversity, estimated using Chao1 and number of OTUs. Taken together, this suggests the number of bacterial taxa in guano does not necessarily change with depth but rather the relative abundances of these taxa within the bacterial community seem to be shifting, allowing certain taxa to dominate the bacterial community as depth of the guano increases.

The PCA revealed clear separation of samples based on depth using weighted UniFrac distances (**Figure 1**). These results further highlight a shift in the relative abundances of the bacterial taxa present within guano with increasing depth. As bat guano is buried and continues to decay, its nutritional composition changes (Bird et al., 2007) and this, along with likely changes in oxygen availability and other environmental factors, may select for a more specialized bacterial community. Specifically, bacterial taxa within the *Enterococcus* and *Bacillus* genera were more abundant as depth of the guano increases (**Figure 2B**). Other genera, such as *Vespertiliibacter* and *Plesiomonas*, decrease in abundance from fresh to surface and subsurface guano (**Figure 2B**), possibly due to either a change in nutritional component present in high abundance within the bat host but no longer available once separated from the host, or a change in environmental conditions necessary for growth. The observation that these genera, particularly *Vespertiliibacter*, were not identified in our cultured isolates indicates the importance of including culture-independent analyses in studies of microbial communities in extreme habitats. Other taxa within the genus *Vespertiliibacter* have also proved challenging for culturing, requiring enrichment culturing techniques (Mühldorfer et al., 2014). In addition, the low variation in relative abundance of functional categories observed using PICRUSt suggests that although relative abundances of taxa are changing as guano goes from being fresh on the surface of a guano pile to deep within the pile, the bacteria being selected for are still performing many of the same core functions.

Although the fresh guano provides the origin for nutrients and microbes for the guano pile, the relative abundances of bacterial taxa found in decaying guano are significantly different than that found in fresh guano (**Figure 1**). We did not characterize the chemical composition of the guano in this study, but we predict that there are biochemical changes in the guano over time and depth in the cave as the result of interactions among coprophagous organisms and microbes. For this study, we only sampled up to 122 cm deep in the pile, but since guano deposit rates are estimated between 2 and 10 cm per year (Hutchinson, 1950), we predict the guano within our sampled cave and other caves could be several meters deep. Furthermore, data indicate that guano characteristics change dramatically after 120 cm depth (Bird et al., 2007), so deeper investigations may yield new bacterial communities.

With our cultured samples, we isolated and identified 14 distinct species, and one potentially new species of bacteria. Four of these species (*Microbacterium oxydans*, *Microbacterium maritypicum*, *Bacillus safensis*, and *Bacillus velezensis*) were found in fresh, surface, and subsurface guano (**Table 3**). These species also grew on all media sources (**Table 3**), suggesting that these bacteria are part of the bat’s microflora and persist throughout the guano pile. The remaining bacteria that were cultured from only one pile location or grew on only one type of agar are likely to be specialists, having more particular nutritional and environmental needs. *Pantoea vagans*, *Staphylococcus lugdunensis*, *Weissella confusa*, *Deinococcus radiopugnans*, and *Streptomyces cremeus* were isolated only from fresh guano, indicating they are restricted in habitat to the gastrointestinal flora of the bats. *Lactococcus garvieae*, *Enterobacter asburiae*, and *Lactococcus lactis* were only isolated from surface guano samples, indicating these species may form specific interactions with the coprophagous insects living on the surface of the guano pile (Bennett and Thies, 2007).

Lastly, specialists present at the subsurface levels, including *Streptococcus rubneri* and the possibly misidentified *Filibacter limicola*, survive and thrive in low-light, low-oxygen conditions present within the guano pile. They are most likely facultatively anaerobic bacteria with flexible nutritional needs that can be accommodated even at increasing depths. Members of the genus *Streptococcus* are known to be facultatively anaerobic; however, this is not the case with *Filibacter*. The genus *Filibacter* contains a single species that is an obligate aerobe (Maiden and Jones, 1984). Although our isolate does not completely match that species (*Filibacter limicola)*, it does appear to match well enough to belong in the same genus. Interestingly, this genus was not identified in our community composition analysis. Further work on this isolate could necessitate emending the description of the genus *Filibacter* to include obligate aerobes as well as facultative anaerobes.

In addition to characterizing the cultured isolates by media type and guano location, we also performed a literature search on the species isolated to determine whether they have been reported to be human pathogens. **Table 3** lists the six species we cultured that are reported as potential human pathogens and that have been cultured from a wide spectrum of clinical specimens including infections of skin and wounds (*Enterobacter asburiae*, Stewart and Quirk, 2001; Koth et al., 2012), the urinary tract (*Microbacterium oxydans*, Gneiding et al., 2008; and *Enterococcus faecalis*, Jett et al., 1994; Richards et al., 2000; Kau et al., 2005), and the cardiovascular system (*Lactococcus garvieae*, Wang et al., 2007; and *Staphylococcus lugdunensis*, Anguera et al., 2005; Frank et al., 2008; and *Weissella confusa*, Olano et al., 2001). These species are found in fresh and surface guano only, suggesting the pathogenic bacteria originate from within the bat gastrointestinal tract, and do not persist when the guano begins to decay. All six of the pathogenic species we identified via culture analysis also match the genera identified from fresh guano from the Indian frugivorous bat (Banskar et al., 2016), further indicating the possibility of similar gastrointestinal bacteria for bats worldwide.

The relatively low numbers of cultured species we obtained is likely explained by the culture media used for isolation, competition from biotypes, and growth conditions. Bacterial isolates obtained by agar-plating are limited in diversity to those microbes which can grow on the selected growth media. For our study, we tested three types of media, TSA, BA, and BGM. Although these three media were chosen to provide diverse nutritional needs, they likely do not encompass the complex nutrients found within the guano pile, which may limit the growth of guano specialists in the laboratory. We did not explicitly characterize the biochemical properties of the guano used in our study, but instead relied on prior published information on the biochemical properties of bat guano (Chroňáková et al., 2009). Furthermore, microbes can be further limited by the abundance at which each species is present in the sample. A “detectable-limit” is therefore created by the most abundant biotypes and/or fastest-growing types that either outcompete or obscure the growth of the less-numerous or slower-growing members in the sample community. Finally, we grew the cultures in aerobic conditions and at 28°C, which may not be indicative of the ambient environment within the guano pile. Future studies on the specific biochemical profile of guano within the pile can lead to the development of more specific BGM, and culturing in anaerobic environments with higher temperatures may lead to the isolation of additional new bacterial species within the guano pile.

Despite the limitations with culturing, the combination of our community composition and culture-based analyses provides new information about the unique bacterial community found within layers of decay in a large bat guano pile. Our data indicate layers within these piles represent unique, extreme microhabitats that dictate different bacterial communities. Evidence from our work can inform future, detailed studies investigating these habitats at different depths. Furthermore, since each cave is a self-contained habitat, and there are numerous large bat caves around the world, there is potential for cave-to-cave variation. Collectively, our results indicate bat caves are under-studied resources for identifying new bacterial species and investigating extreme microbiology habitats.

## Author Contributions

MN performed the culture-independent data analysis, interpreted the data, and wrote the manuscript. LK collected the samples, performed the culture analysis, interpreted the data, and wrote the manuscript. MD performed the culture analysis and wrote the manuscript. JM performed the culture analysis, interpreted the data, and wrote the manuscript. JK supervised all the aspects of the project and wrote the manuscript.

## Conflict of Interest Statement

The authors declare that the research was conducted in the absence of any commercial or financial relationships that could be construed as a potential conflict of interest. The reviewer FH and handling Editor declared their shared affiliation.

## Footnotes

1 http://www.mrdnalab.com/

2 http://rdp.cme.msu.edu

3 www.ncbi.nlm.nih.gov

## Supplementary Material

TABLE S1 Components of the bat guano medium (BGM).

TABLE S2 Operational taxonomic units (OTUs) that differed significantly among our three sampling locations. Significant difference was determined using a non-parametric two-sample *t*-test with Monte Carlo permutations (α = 0.05).
