# Supplementary material for: Variation in Bat Guano Bacterial Community Composition With Depth
Source: Front Microbiol. 2018 May 11;9:914. doi: 10.3389/fmicb.2018.00914 (PMC5958644; doi:10.3389/fmicb.2018.00914)
Supplement: TABLE S1 — Components of the bat guano medium (BGM). [file Table_1.DOCX]

Supplementary Material

Supplementary Table 1: Components of the Bat Guano Medium (BGM)

| **Ingredient                                                      Quantity** | |
| --- | --- |
| Na_2_HPO_4_ • 7H_2_O dibasic  KH_2_PO_4_ dibasic  NaCl  NH_4_Cl  MgSO_4_  CaCl_2_  glucose  galactose  lactose  sucrose  mannitol  agar  water | 12.8 g  3.0 g  0.5 g  7.5 g  0.24 g  0.011 g  3.5 g  3.5 g  3.5 g  3.5 g  3.5 g  18.0 g  1 L |
